# Hybrid electroconvulsive therapy in an adolescent with major depressive disorder: a case report

**DOI:** 10.3389/fpsyt.2024.1487983

**Published:** 2025-01-17

**Authors:** Jing-ya Zhang, Jia Li, Nan Wang, Xin-hui Xie, Lun Zeng

**Affiliations:** ^1^ Department of Clinical Psychology, The Second People’s Hospital of Huizhou, Huizhou, Guangdong, China; ^2^ Electroconvulsive Therapy Room, Department of Psychosomatic Medicine, The Second People’s Hospital of Huizhou, Huizhou, Guangdong, China; ^3^ Department of Child & Adolescent Psychology, The Second People’s Hospital of Huizhou, Huizhou, Guangdong, China; ^4^ Brain Function and Psychosomatic Medicine Institute, The Second People’s Hospital of Huizhou, Huizhou, Guangdong, China; ^5^ Department of Psychiatry, Renmin Hospital of Wuhan University, Wuhan, Hubei, China

**Keywords:** electroconvulsive therapy, MDD, adolescent, HECT, cognitive function

## Abstract

The incidence of depression is increasing in adolescents, who are at a stage of education and therefore more concerned about their cognitive changes. We tried to preserve the rapid relief of depressive symptoms in electroconvulsive therapy (ECT) while causing less cognitive impairment, thus designing the hybrid-ECT (HECT), a modified ECT therapy. Here, we report a case study of a 14-year-old male student with major depressive disorder (MDD) suffering from severe suicidality and significant impairment in social functioning who achieved effective antidepressant effects with HECT and improved cognitive function. HECT showed safety and effectiveness in adolescent MDD patient.

## Introduction

ECT is a rapid and effective treatment for MDD, with a response rate of 60-80% (1). However, clinicians are hesitant to use it due to its cognitive adverse effects, particularly in children and adolescents, who are more cautious than adults with MDD. To address this issue, our team designed a modified ECT therapy- hybrid-ECT (HECT), in which the first three sessions use ECT, and subsequent sessions use low-charge electrotherapy (LCE), shown good antidepressant efficacy and better cognitive performance in RCT studies of depression in adults (2). However, the impact of HECT on adolescent patients is still unknown. Here, we present this case of a 14-year-old male MDD patient with persistent self-injurious behavior and severe suicidal ideation who achieved favorable antidepressant effects and better cognitive performance after HECT treatment.

## Case presentation

A 14-year-old right-handed male student with a 6-month history of depression. From February 2023, he began to feel depressed, lack energy, social withdrawal, headaches, dream a lot and wake up early, and irritability. Gradually, he began to scratch his arm with knives, which later progressed to hurting himself every day and developing increasingly suicidal thoughts. In July 2023, he visited a psychiatric outpatient clinic and took fluoxetine 20mg and dexzopiclone 2mg daily, but stopped taking those medication due to diarrhea after 3 days. In August 2023, he was hospitalized in the Second People’s Hospital of Huizhou because of intense suicidal thoughts, worse mood, loss of confidence in learning and socializing, spend most of his time lying in bed worrying about how to get through each day, anhedonia and withdrawal from school. His parents reported that he had normal physical and mental development, excellent academic performance, and no recent stress events. According to the *International Classification of Diseases, Tenth Revision*, criteria, the patient was diagnosed with a major depressive episode without psychotic features, had escitalopram 10 mg (the dose was increased after 4 days to 20 mg) and zopiclone 7.5 mg once a day from August 3rd. His parents found it increasingly difficult to manage the patient’s self-injurious behaviors and demand rapid relief of his depressive symptoms, as well as the patient met the indications for ECT, so we decided to proceed with ECT treatment.

Because of the good therapeutic effect of HECT in adult clinical trials, HECT treatment was performed after consultation with the patient and his parents, they were fully informed and signed informed consent. In HECT, the first three sessions consist of standard bilateral ECT (energy set at 1.5 times the seizure threshold (ST)). Starting from the fourth session, it transitions to LCE, distinguished from ECT by dosing at only 0.5 times the ST without monitoring whether a seizure occurs. In general, ECT/LCE procedures were performed using a spECTRUM 5000Q ECT instrument (MECTA Corporation, OR, United States), with a pulse width of 1 ms and a fixed current of 800 mA. A dose titration procedure was performed to determine the ST at the first session.

Before HECT, laboratory and physiological examinations showed that blood-test results (thyroid function, electrolytes, cortisol), liver and kidney function were normal, and the heart rate, as well as head computed tomography (CT) examinations, revealed no obvious abnormalities. Symptom severity was assessed with the Montgomery-Åsberg Depression Rating Scale (MADRS) with a score of 38 (severe depression). The self-rating scales were the 9-item Patient Health Questionnaire (PHQ-9) and the Generalized Anxiety Disorder 7 (GAD-7). Cognitive functions were tested using the Everyday Memory Questionnaire (EMQ), Repeatable Battery for the Assessment of Neuropsychological Status (RBANS), and the Stroop Color and Word Test.

**Figure 1:**
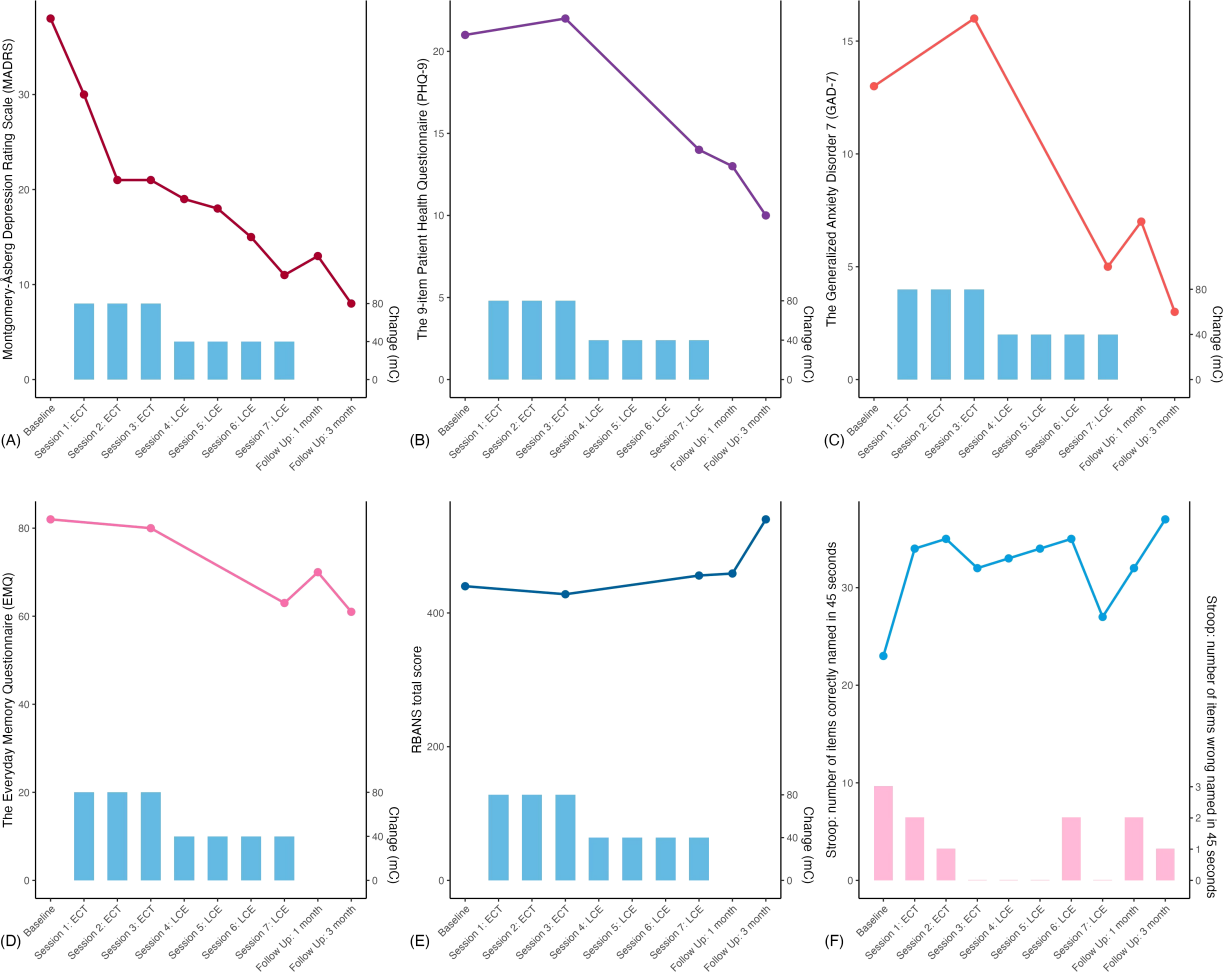
Comprehensive results of the patient. The x-axis in all panels marks the number of follow-up visits, and the blue vertical bar charts represent the electricity setting of the treatment. **(A)** the Montgomery-Åsberg Depression Rating Scale (MADRS); **(B)** the 9-item Patient Health Questionnaire (PHQ-9); **(C)** the Generalized Anxiety Disorder 7 (GAD-7); **(D)** the Everyday Memory Questionnaire (EMQ); **(E)** the Assessment of Neuropsychological Status (RBANS) and **(F)** the Stroop Color and Word Test, the dashed line indicates the number of correct readings within 45 seconds, and the pink vertical bar charts represent the number of incorrect readings within 45 seconds.

The patient received a total of 7 sessions of the bitemporal electrode placement treatments (three times per week), between August 5 and August 24, 2023. The specific charge setting and seizure durations for ECT and LCE were shown in **Figure 1**. Anesthetic drug was 30 mg of etomidate, muscle relaxation with succinylcholine (70 mg) and atropine (0.5 mg). After the first treatment, the patient reported a headache, which was relieved by ibuprofen 0.1g.

The five visits were set at (1) baseline; (2) post-ECT sessions; (3) post-LCE sessions; (4) end of the 1-month follow-up period; and 5) end of the 3-month follow-up period. In addition, MADRS and the Stroop test were performed after each session. After the end of all treatments, the MADRS score was 11 (achieved remission), and the antidepressant effect of HECT was maintained at 3 months of follow-up. All detailed results were shown in **Figure 1**.

## Discussion

The adolescent depressed patient showed good antidepressant effect with the HECT protocol, achieving clinical remission, and was maintained at a 3-month follow-up. The patient’s cognitive performance was also better than the baseline after the end of treatment.

Adolescent depression is becoming increasingly common, 34% of adolescents globally (aged 10-19 years) are at risk of developing clinical depression (3). Severe depressive symptoms and a high risk of suicide in children and adolescents can have a significant influence on their functioning, early and effective treatment can help shorten the duration of the disease, reducing time away from school, friends, and family. In this case, HECT was utilized for the first time in adolescent MDD. According to the trajectory of MADRS scores, the score reduction rate of the patient after the second ECT treatment was close to 50%, and the trajectory gradually flattened after that. Combined with the ECT treatment of adults with depression by other researchers, the trajectory of adults’ data shows that even if about a 10-point reduction after only one ECT session, the rate of depression score reduction is more rapid in the first 3-5 times, and then gradually flattens (4, 5). The adolescent patient experienced a headache only after the first ECT treatment, which was relieved after ibuprofen, showing that HECT has sufficient safety.

In terms of the cognitive function assessment, the trajectory of EMQ score reduction was similar to that of MADRS, which showed that the memory function of the patient improved with the reduction in symptom severity. Previous studies have shown that the most common side effect reported by depressed adolescents receiving ECT is subjective memory complaint, and this was also the main reason for restricted treatment (6). The scores of RBANS and STROOP tests were higher than the baseline after HECT, suggesting that the cognitive and executive functions of this patient were improved. The results are similar to our previous study on adult MDD patients (2), indicating that HECT can also achieve better cognitive outcomes for adolescents with depression.

ECT is a safe and effective treatment for major depression. The response rate of regular ECT was about 60%-80% and the remission rate was about 35% (7), while the HECT in adult MDD patients of RCT results showed the response rate and remission rate were 73.3% and 26.7%, respectively (2). In addition, it is worth noting that the results of RCT showed that the adverse events in the LCE stage was significantly less than that in the control group treated with regular ECT. A retrospective study by the Mayo Clinic found that in the adolescent population, depression had an ECT response rate of 63% (8). However, both professional and lay groups continue to oppose and stigmatize the use of ECT in children and adolescents, creating barriers to pediatric ECT availability. The HECT protocol offers promise as a rapid antidepressant treatment that combines the advantages of ECT in terms of immediate and effective reduction of depressive symptoms and LCE, as well as safety and limited cognitive impairment.

In summary, the HECT showed rapid antidepressant effects for depressed adolescents with severe suicide risk and impaired functioning, with the added benefit of improving cognitive functions. We need a larger sample size to test whether HECT can be a better treatment option for adolescents with MDD.

## Data Availability

The raw data supporting the conclusions of this article will be made available by the authors, without undue reservation.
