# Supplementary material for: Hybrid electroconvulsive therapy in an adolescent with major depressive disorder: a case report
Source: Front Psychiatry. 2025 Jan 17;15:1487983. doi: 10.3389/fpsyt.2024.1487983 (PMC11783184; doi:10.3389/fpsyt.2024.1487983)
Supplement: Supplementary file 1 [file Supplementaryfile1.pdf]

CARE case report guidelines

CARE Checklist of information to include when writing a case report

BY-NC-ND

Topic	Item	Checklist item description	Reported on sections/paragraphs
Title	1	The diagnosis or intervention of primary focus followed by the words "case report"	section "article title"
Key Words	2	2 to 5 key words that identify diagnoses or interventions in this case report, including "case report"	section "keywords"
Abstract (no references)	3a	Introduction: What is unique about this case and what does it add to the scientific literature?	section "abstract"
	3b	Main symptoms and/or important clinical findings	section "abstract"
	3c	The main diagnoses, therapeutic interventions, and outcomes	section "abstract"
	3d	Conclusion—What is the main "take-away" lesson(s) from this case?	section "abstract"
Introduction	4	One or two paragraphs summarizing why this case is unique (may include references)	section "introduction"
Patient Information	5a	De-identified patient specific information.	section "case presentation" paragraph 1
	5b	Primary concerns and symptoms of the patient.	section "case presentation" paragraph 1
	5c	Medical, family, and psycho-social history including relevant genetic information	section "case presentation" paragraph 1
	5d	Relevant past interventions with outcomes	section "case presentation" paragraph 1
Clinical Findings	6	Describe significant physical examination (PE) and important clinical findings.	section "case presentation" paragraph 3
Timeline	7	Historical and current information from this episode of care organized as a timeline	FIGURE 1
Diagnostic Assessment	8a	Diagnostic testing (such as PE, laboratory testing, imaging, surveys)	section "case presentation" paragraph 3
	8b	Diagnostic challenges (such as access to testing, financial, or cultural)	NA
	8c	Diagnosis (including other diagnoses considered)	section "case presentation" paragraph 1
	8d	Prognosis (such as staging in oncology) where applicable	NA
Therapeutic Intervention	9a	Types of therapeutic intervention (such as pharmacologic, surgical, preventive, self-care)	section "case presentation" paragraph 2
	9b	Administration of therapeutic intervention (such as dosage, strength, duration)	section "case presentation" paragraph 2,4
	9c	Changes in therapeutic intervention (with rationale)	section "discussion" paragraph 4
Follow-up and Outcomes	10a	Clinician and patient-assessed outcomes (if available)	FIGURE 1
	10b	Important follow-up diagnostic and other test results	FIGURE 1
	10c	Intervention adherence and tolerability (How was this assessed?)	section "case presentation" paragraph 4,5
	10d	Adverse and unanticipated events	section "case presentation" paragraph 4
Discussion	11a	A scientific discussion of the strengths AND limitations associated with this case report	section "discussion" paragraph 1
	11b	Discussion of the relevant medical literature with references.	section "discussion" paragraph 2,3,4
	11c	The scientific rationale for any conclusions (including assessment of possible causes)	section "discussion" paragraph 2,3,4
	11d	The primary "take-away" lessons of this case report (without references) in a one paragraph conclusion	section "discussion" paragraph 5
Patient Perspective	12	The patient should share their perspective in one to two paragraphs on the treatment(s) they received	NA
Informed Consent	13	Did the patient give informed consent? Please provide if requested	Yes ☑ No ☐
